# Translocator Protein-Mediated Stabilization of Mitochondrial Architecture during Inflammation Stress in Colonic Cells

**DOI:** 10.1371/journal.pone.0152919

**Published:** 2016-04-07

**Authors:** Leeyah Issop, Mariano A. Ostuni, Sunghoon Lee, Mireille Laforge, Gabriel Péranzi, Pierre Rustin, Jean-François Benoist, Jérome Estaquier, Vassilios Papadopoulos, Jean-Jacques Lacapère

**Affiliations:** 1 Sorbonne Universités – Université Pierre et Marie Curie Université de Paris VI, École Normale Supérieure – PSL Research University, Département de Chimie, CNRS UMR 7203 LBM, 4 Place Jussieu, F-75005, Paris, France; 2 The Research Institute of the McGill University Health Center and the Department of Medicine, McGill University, Montreal, Quebec, H4A 3J1, Canada; 3 INSERM UMRS 1134, Institut National de la Transfusion Sanguine, 6 rue Alexandre Cabanel, Université Paris 7 Denis Diderot, F-75015 Paris, France; 4 CNRS FR 3636, Université Paris Descartes, Paris, France; 5 INSERM UMR 1141, Hôpital Robert Debré, and Université Paris 7 Denis Diderot, F-75019, Paris, France; 6 Université Laval, Faculté de Médecine, Département de microbiologie-infectiologie et d’immunologie, Quebec City, Quebec, G1V06A, Canada; Roswell Park Cancer Institute, UNITED STATES

## Abstract

Chronic inflammation of the gastrointestinal tract increasing the risk of cancer has been described to be linked to the high expression of the mitochondrial translocator protein (18 kDa; TSPO). Accordingly, TSPO drug ligands have been shown to regulate cytokine production and to improve tissue reconstruction. We used HT-29 human colon carcinoma cells to evaluate the role of TSPO and its drug ligands in tumor necrosis factor (TNF)-induced inflammation. TNF-induced interleukin (IL)-8 expression, coupled to reactive oxygen species (ROS) production, was followed by TSPO overexpression. TNF also destabilized mitochondrial ultrastructure, inducing cell death by apoptosis. Treatment with the TSPO drug ligand PK 11195 maintained the mitochondrial ultrastructure, reducing IL-8 and ROS production and cell death. TSPO silencing and overexpression studies demonstrated that the presence of TSPO is essential to control IL-8 and ROS production, so as to maintain mitochondrial ultrastructure and to prevent cell death. Taken together, our data indicate that inflammation results in the disruption of mitochondrial complexes containing TSPO, leading to cell death and epithelia disruption. *Significance*: This work implicates TSPO in the maintenance of mitochondrial membrane integrity and in the control of mitochondrial ROS production, ultimately favoring tissue regeneration.

## Introduction

Inflammatory bowel diseases (IBD) are characterized by chronic relapsing inflammation of the gastrointestinal tract due to a deregulated mucosal immune response. The cascade of events leading to the development of IBD remains poorly understood [1,2]. Notably, IBD patients are at risk of developing cancer [3]. This association is thought to arise from the existence of an inflammation–dysplasia–carcinoma sequence [4].

The translocator protein (18 kDa; tryptophan-rich sensory protein oxygen sensor, TSPO), previously known as peripheral-type benzodiazepine receptor, is a protein mainly located in the outer mitochondrial membrane of most cell types [5]. TSPO is implicated in many physiological functions, such as steroidogenesis, cell proliferation, and apoptosis. Its expression not only varies according to tissue type, but also between healthy and pathological tissues. TSPO is expressed in the healthy colon and overexpressed in colon cancer [6–11]. We previously showed that TSPO is also overexpressed in IBD, such as ulcerative colitis and Crohn’s disease, as well as in dysplasia [11]. TSPO overexpression was induced in an animal model of IBD and high-affinity TSPO drug ligands were shown to regulate cytokine production and improve tissue reconstruction [11]. Despite these observations, the intracellular mechanisms implicated in these processes are not known and may be important not only for understanding normal colon function, but also in unveiling the inflammation–dysplasia–carcinoma sequence in colonic diseases.

The HT-29 human colon carcinoma cell line has been described as a model of intestinal epithelial cells [12] expressing TSPO [13]. Tumor necrosis factor (TNF) treatment of these cells has effects similar to those observed with freshly isolated epithelia cells [14]. It activates several cellular pathways, regulating the expression of various proteins and the secretion of a number of proinflammatory factors [15]. We therefore used this colon cell line to study TSPO regulation and TSPO drug ligand effects upon TNF-induced inflammation to characterize intracellular mechanisms involved in this process.

The results obtained indicate that TNF-induced TSPO overexpression occurs later than the induction of interleukin (IL)-8 expression, which is coupled to reactive oxygen species (ROS) production. This inflammatory process leads to mitochondrial hyperfusion, which was previously described as an adaptation to stress [16]. We also report that initial TNF-induced changes of mitochondrial cristae can be reversed and/or prevented by treatment with a TSPO drug ligand. Taken together, these data suggest that the mitochondrial disruption occurring during inflammation might lead to cell death and epithelial disruption, similar to what is observed in IBD [11]. Exposure to TSPO drug ligands for a short time of inflammation maintains the mitochondrial ultrastructure and thus reduces inflammation through ROS regulation, which could be responsible of tissue regeneration. We conclude that TSPO is an architectural protein essential for maintaining the mitochondrial ultrastructure.

## Materials and Methods

### Cell culture

The human colonic adenocarcinoma cell line HT-29 (passages 166–170) were obtained from Dr C.L. Laboisse (Medical School, 44035 Nantes, France). Cells were grown at 37°C in a 5% CO_2_ atmosphere in culture medium (Dulbecco’s Modified Eagle’s Medium with Glutamax; Gibco^®^, Thermo Fisher Scientific, Invitrogen Corporation, France) and supplemented with 10% fetal calf serum, as described previously [13,17]. Cells were initially plated at 3×10^5^/cm^2^ and they were cultured for 48 hours to enable cell adhesion; then, the culture medium was removed and replaced with fresh medium containing TNF-alpha (TNF-α) alone (10 mg/mL unless specially indicated; R&D Systems Europe, Lille, France) or with the high-affinity ligand PK 11195 (20 mM stock solution in ethanol; Sigma-Aldrich Co., Saint-Quentin Fallavier, France) used at a concentration of 1 μM. The inflammation treatments vary from a few hours to 4 days; the treatment was renewed every day.

### Proliferation

Cell proliferation was quantified using the crystal violet assay. Briefly, after removing the supernatant, cells were washed with phosphate buffered saline (PBS; 150 mM NaCl, 50 mM PO_4_NaH, pH 7.4; Sigma-Aldrich Co.) and fixed with 70% ethanol solution. Then, cells were incubated with crystal violet solution with a final concentration of 0.08% for 5 minutes with stirring. The cells were washed and incubated with acetic acid 33% for 1 minute upon stirring. The optical density was red at a wavelength of 570 nm using the multilabel plate reader (Victor^™^; PerkinElmer Inc., Villebon sur yvette, France); quantification was performed using standard calibration with a known number of cells.

### Flow cytometry

#### Cytokines

Cell culture medium was collected at the indicated time following incubation in the different conditions (control, TNF, PK 11195, and TNF + PK 11195) and it was kept at –20°C up until the time of measurement. Proinflammatory cytokine quantification (IL-1b, IL-6, IL-8, IL-10, IL-12p70, and TNF) was achieved using the BD cytometric array kit “BD Cytometry Array”, and it was read by the BD FACSCalibur^™^ cytometer (BD Biosciences, Le Pont de Claix, France). For the specific study on IL-8, the detection and quantification of the cytokines were performed using IL-8 ELISA Quantikine (R&D Systems Europe) and in a multilabel plate reader.

#### Apoptosis

Cells were harvested and fixed in ethanol (70%) at the indicated time after incubation in the different conditions (control, TNF, PK 11195, and TNF + PK 11195). Cells were washed in PBS and incubated for 30 minutes at room temperature in the presence of RNAse A (100 μg/mL; Sigma-Aldrich Co.). Propidium iodide (PI; Sigma-Aldrich Co.) was added at a final concentration of 75 μg/mL and kept in the dark for 15 minutes at 37°C. PI fluorescence was measured by flow cytometry (Beckman Coulter Inc., Villepinte, France) with a laser excitation wavelength set at 620 nm and an emission pass band set at 488–540 nm.

Quantification of apoptosis was made using the AnnexinV–FITC kit (Roche Diagnostics, Meylan, France). Cells were grown in 12-well plates and harvested after the different treatments. Fluorescence was measured with the laser excitation wavelength set at 488 nm and an emission wavelength set at 518 nm using flow cytometry (Beckman Coulter Inc.). Quantitative analysis was performed using the EXPO 32 software (Beckman Coulter Inc.). PI was used as a control for the necrotic cells.

### Transcript and Protein expression

#### RTqPCR

HT-29 cells were harvested by adding the trypsin replacement enzyme (TriplE; Thermo Fisher Scientific) at the indicated time after incubation in the different conditions (control, TNF, PK 11195, and TNF + PK 11195). Cell pellets (obtained by centrifugation at 1500 g for 10 minutes) were treated with the RNeasy plus Kit (Qiagen SAS, Courtaboeuf, France) to extract mRNA, which was measured with Nanodrop (Labtech International Ltd, Palaiseau, France) and kept at –80°C until use. Samples were normalized to the total RNA content and reverse-transcribed using the Verso^™^ cDNA Kit (Thermo Fisher Scientific). The resulting cDNA samples were kept at –20°C. cDNA samples were diluted with nuclease-free water and subjected to real-time quantitative reverse transcription polymerase chain reaction (RTqPCR) using the SYBR Green dye SYBR Green I master mix (Roche Diagnostics) technique and quantified with the Light Cycler 480 (Roche Diagnostics); the primers corresponded to the chosen genes (*TSPO*, *IL-8*, *Mfn1/2*, and *OPA1*). Measured cycle thresholds for the different mRNAs were normalized to *GAPDH*, which was used as an endogenous control [18]. The oligonucleotide sequences of the sense and antisense primers used were QT01173921 for *TSPO*, QT00000322 for *IL-8*, QT00077966 for *Mfn1*, QT00057589 for *Mfn2*, and QT00085519 for *OPA1*, all purchased from Qiagen.

#### Immunoblots

Protein samples were layered on top of sodium dodecyl sulfate (SDS)–polyacrylamide gel electrophoresis (PAGE), and they were electrophoretically separated on a 4%–12% Tris-glycine SDS-PAGE. Proteins were transferred on polyvinylidene fluoride membranes and blocked for 90 minutes at room temperature (20 mM Trizma Base; 100 mM NaCl; 1% Tween-20; 5% skim milk). Membranes were hybridized with monoclonal antibodies overnight at 4°C with primary antisera against TSPO (Trevigen Inc., Interchim, Montlucon, France). Membranes were washed and incubated for 1 hour at room temperature with secondary antirabbit immunoglobulin (Ig)G horseradish peroxidase (HRP)-linked antibody (Cell Signaling Technology, Inc., Ozyme, Saint-Quentin, France). The proteins of interest were visualized using the Amersham chemiluminescence kit (GE Healthcare, Velizy, France) and a FUJI image reader LAS4000 (Fujifilm, GE Healthcare, Velizy, France) to capture images. Several antibodies were used sequentially on the same membrane (TSPO, OPA1, Actin, SLP-2, and Mfn). Gel quantification analysis was performed using the ImageJ software.

### Mitochondria isolation

HT-29 mitochondria were isolated as previously described [13]. Briefly, cells were scrapped and harvest from three 75 cm^2^ flasks. Cells were centrifuged, and pellets were potterized and submitted to three cycles of freeze and thaw. Whole homogenate was centrifuged at 1200 g for 10 minutes. The supernatant was centrifuged at 11000 g for 20 minutes. The resulting pellet was enriched in mitochondrial membranes. The isolation of mitochondrial complexes and supercomplexes was achieved using 0.1% (w/v) dodecylmaltoside (DDM) pr 0.1% (w/v) digitonin (Digit) solubilization, as previously described [19].

### Biochemical methods

#### Oxygraphy

HT-29 cells were harvested, centrifuged, washed in PBS buffer, and kept on ice until the point of measurement in a 3 mL thermos-jacketed sample chamber containing a Clark oxygen electrode (Hansatech Instruments, Cergy, France), as previously described [20].

#### Lactate and pH assay

HT-29 cell supernatant was collected at the indicated incubation time, and lactate content was measured by gas chromatography–mass spectrometry (Varian Saturn-2000; Agilent Technologies, les Ulis, France). The pH assay was performed upon pooling of the cell supernatant (1 mL or 2 mL from several wells) collected after 24 hours of cell culture.

#### Glutathione

Glutathione was extracted from HT-29 cells by osmotic lyses and protein acid precipitation using a final concentration of SSA 5%. Total glutathione (GSH and GSSG), as well as reduced GSSG, were measured by spectrophotometer [21], using NADPH and glutathione reductase with or without DTNB at 412 nm and 340 nm, respectively.

### Imaging studies

#### Electron microscopy

Following the different treatment conditions, cells were directly fixed in situ in a 25 cm^2^ flask, embedded in epon resin, and sections were obtained as previously described [22]. Sections were viewed in a JEOL 1200 EX electron microscope at an accelerated voltage of 80 kV. Low magnification reveals cells and high magnification permit to observed mitochondria and to analyze mitochondrial morphology. Three type of morphology were defined: type 1 (well formed cristae, appearing as stacked and parallel membrane), type 2 (mixture of well formed cristae and circular-appearing internal membranes) and type 3 (circular-appearing internal membranes). For each conditions (Control, TNF-treated with or without PK 11195 and PK 11195 alone) several sections were observed and mitochondria analyzed in each cell for classification in either type. When the internal structure was not clearly observable, mitochondria were not taken into account.

#### Stress fibers (actin staining)

The cell culture medium was removed and HT-29 cells were washed three times with PBS buffer. Cells were fixed with formalin solution 2% (Sigma-Aldrich Co.) for 10 minutes. Then, cells were washed three times, firstly with PBS buffer containing 0.1% triton, and secondly with PBS buffer containing 1% bovine serum albumin (BSA). Phalloidin–rhodamin was added to the cells and were air dried and mounted (vectashield) with cover slips and observed with a Zeiss confocal microscope (541 nm excitation wavelength and 575 nm emission wavelength).

#### Analysis of mitochondrial network

HT-29 cell lines have been transfected by a lentivirus containing a kindly donated green fluorescent protein (GFP) plasmid (by Muriel Priault). HT-29 cells transfected with Mito-GFP were imaged at the indicated time after incubation in the different conditions (control, TNF, PK 11195, and TNF + PK 11195). Images were acquired with a videomicroscope (equipped with a x100 oil immersion objective; Leica^™^, Leica Microsystems SAS, Nanterre, France).

Images were analyzed using ImageJ (1.46r) software and following the different steps previously described [23]: (“Brightness and Contrast”); application of a specific filter associated with a “specific mathematic” type “Koopman” with the functions “Convolve” and “make binary”; acquisition and treatment of the different parameters with “analyze particle”); and the final data were analyzed and collected using the Microsoft Excel program (Microsoft France, Issy-les-Moulineaux, France).

For the morphology, two parameters were calculated from the treated data: “AA” for the “Aspect Ratio”, which is the ratio between the major and minor axis of the object considered as an ellipse; and “FF” for “Form Factor” (perimeter^2^/4π × surface), which determines the length and degree of branching. These two parameters, independent of the magnification of the image, have a minimum value of 1 corresponding to a circular shape of the mitochondria. Statistical tests were performed to compare the obtained images according to different times and treatments.

### Transfection

#### TSPO SiRNA

Cells were plated onto 12-well plates at an initial concentration of 3×10^5^ cells per well, and immediately transfected using the TriFECTa^®^ kit DsiRNA duplex (Integrated DNA Technologies, Inc., Coralville, IA, USA) using Lipofectamine^™^ RNAiMAX (Thermo Fisher Scientific).

The following small-interfering (si)RNA duplexes (50 nM) used for TSPO (NM_000714) were: duplex 1,5- CGA CCA CAC UCA ACU ACU GCG UATG-3; duplex 2,5-ACG CUU UCA UGA CCA CUG GGC CUG C -3; and duplex 3,5-UAC GGC UCC UAC CUG GUC UGG AAA G -3. Gene expression and target gene knockdown were evaluated by RTqPCR. After 48 hours, cells were treated (or not) with TNF at different time points. In these different conditions, mock and scrambled were used as controls.

#### TSPO cDNA

Total RNA from HT-29 cells was isolated using the RNASE KIT and it was reverse transcribed. Human cDNA of TSPO was obtained using polymerase chain reaction (PCR) amplification from HT 29 cDNA (polymorphism sequenced as A147, R162). The TSPO cDNA coding sequence was gel purified, ligated, and reinserted into the following plasmid: PCDNA 3.1/HYGRO EGFP (Clontech Laboratories, Inc., Otsu, Shiga, Japan) at the Nhe I and Xho I sites. The constructs were verified by sequencing.

Cells were transfected with human TSPO cDNA and plated into 12-well plates at an initial concentration of 3×10^5^ cells per well. Following 48 hours of transfection, the cell medium was replaced with fresh medium with or without TNF. Gene expression and target gene knockdown were evaluated by RTqPCR. In these different conditions, mock and scrambled were used as controls.

### Statistical analysis

In each graph, unless otherwise noted, the data represent the mean ± standard error of the mean. If indicated, statistical significance has been calculated by one-way analysis of variance followed by Dunnett’s multiple comparison tests. Differences were considered significant when P<0.05. Statistical differences of the mitochondrial ultrastructure's type were analyzed using a contingency chi-square test.

## Results

### Cytokine production

**Fig 1:**
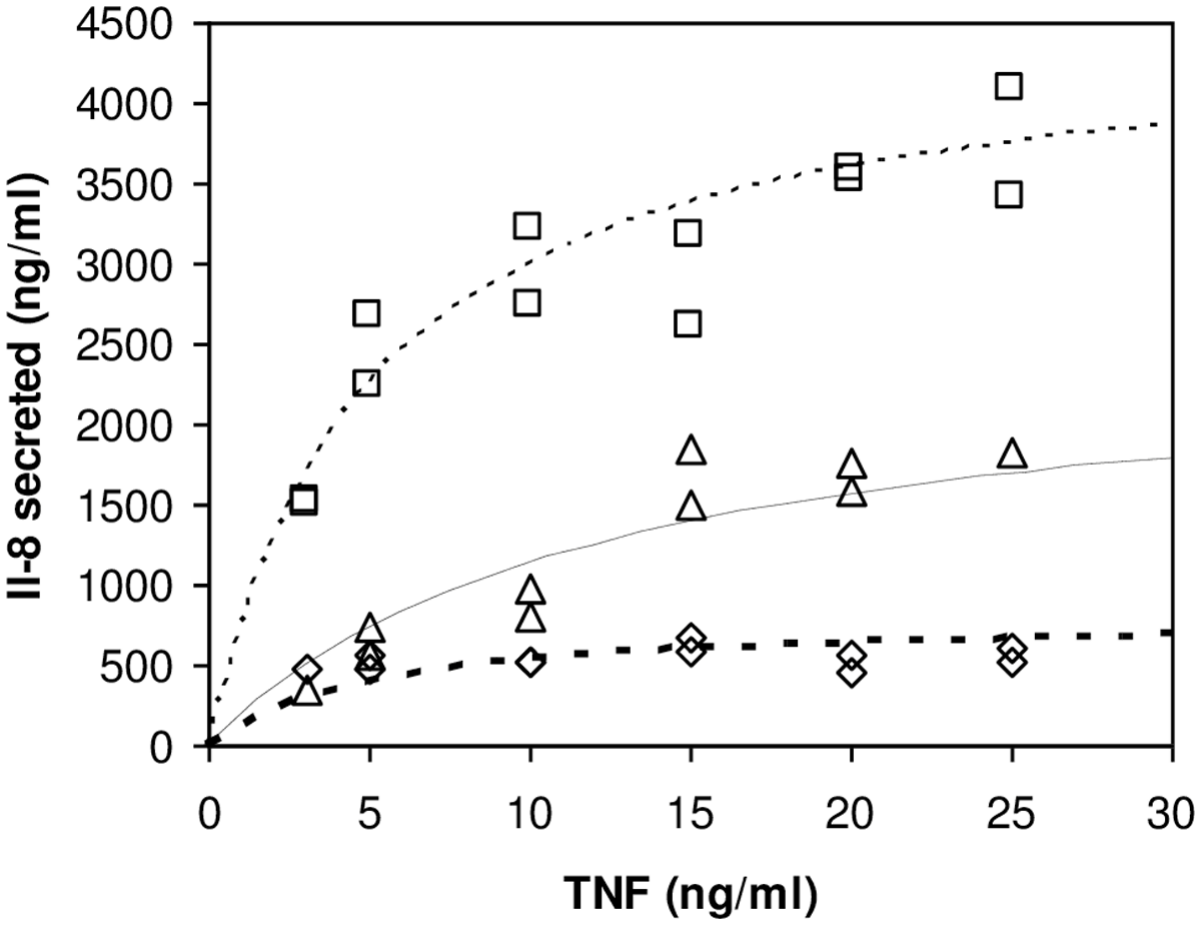
Interleukin-8 production by TNF-treated HT-29 cells. TNF concentration of proinflammatory cytokine IL-8 production after 6-hour [diamonds], 14-hour [triangles], or 24-hour [squares] treatment. The results are expressed as the minimum and maximum values of triplicate of at least 3 independent experiments.

**Fig 2:**
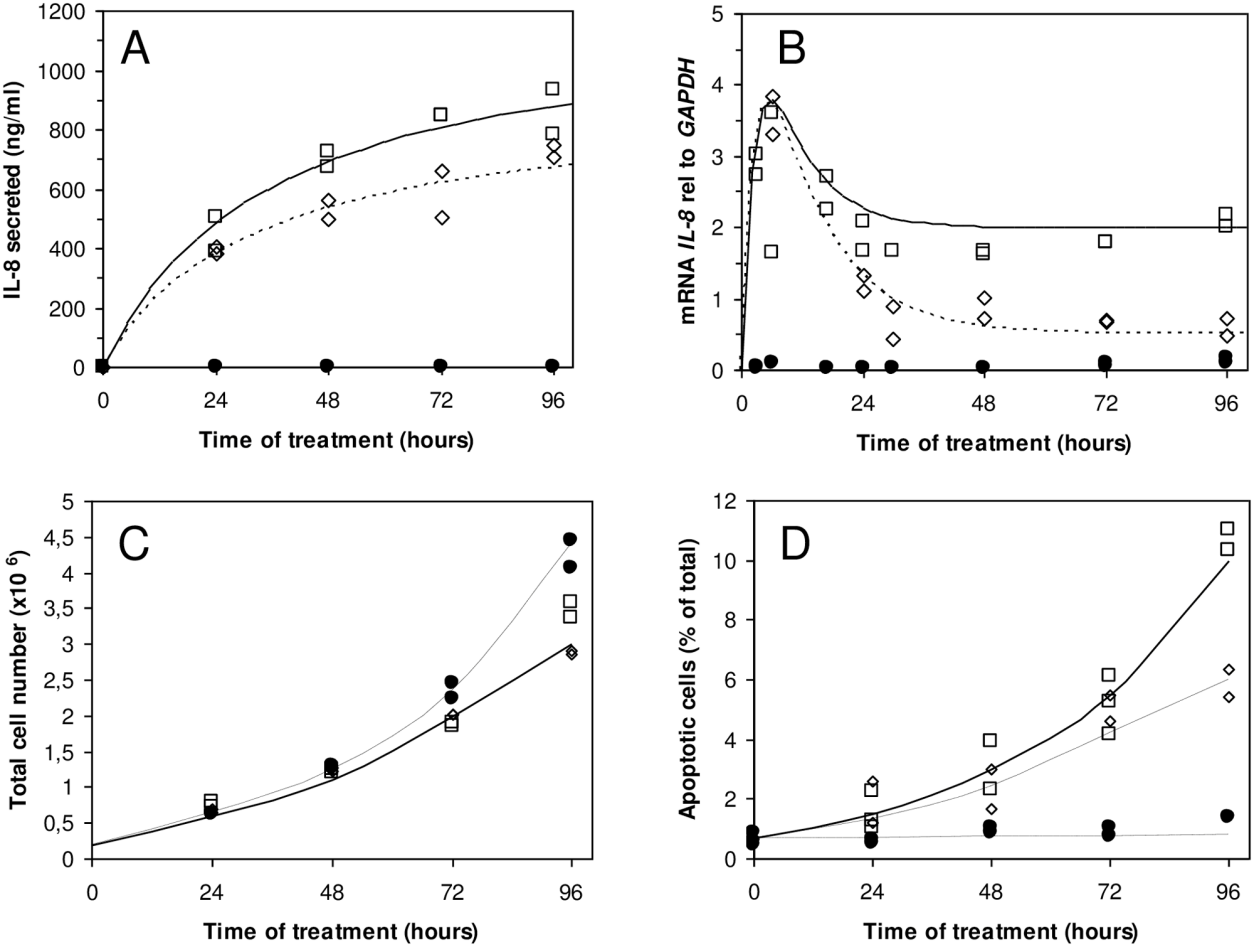
Inflammation in HT-29 cells treated by TNF. A, time course for 10 ng/mL daily repeated treatment. *IL-8* mRNA expression relative to *GAPDH* as a function of time of treatment with 10 ng/mL of TNF, replaced daily (B). Cell proliferation (C) and apoptosis (D) as a function of time (closed circles, control; open squares, 10 ng/of mL TNF replaced daily; and open diamonds, 10 ng/mL of TNF and 1 μM of PK 11195, replaced daily). The results are expressed as the minimum and maximum values of triplicate of at least 3 independent experiments.

Treatment of HT-29 human colon carcinoma cells by TNF-α for 24 hours induces the secretion of the proinflammatory cytokine IL-8 in a dose-dependent manner (Fig 1). This secretion is time dependent since at 6 hours and 14 hours this secretion is extremely low in agreement with previous studies [24]. As a result, in order to perform long-term exposure to TNF, we used the moderate treatment of HT-29 cells with 10 ng/mL of daily renewed medium [25]. Fig 2A shows that IL-8 secretion saturates and does not increase as expected for cells with a doubling rate of about 24 hours. It can reflect a progressive change of physiological cell state, when cells tend to confluency. Interestingly enough, adding a TSPO’s high-affinity drug ligand, PK 11195, in the medium with TNF significantly reduces IL-8 secretion (open diamonds in Fig 2A, p<0.05), further increasing the tendency of IL-8 secretion to saturate. The levels of mRNA for *IL-8* determined by RTqPCR reach their maximum within the first 24 hours with a four-fold increase when compared to non-treated cells (black circles in Fig 2B); they then remain constant for the following days. The presence of PK 11195 does not modify the maxima observed after 6 hours of TNF treatment, but it greatly lowers the values of *IL-8* mRNA observed from 24–96 hours (p<0.001). We analyzed the proliferation of HT-29 cells and observed that culture in the presence of low doses of TNF (Fig 2C) shows only a small decrease in the total cell number after 96 hours. This is in agreement with previous studies [25] showing that HT-29 cells can be exposed for up to 3 weeks to low doses of TNF with different effects from acute exposure to TNF. For instance, acute treatment can lead to early cell death [15], whereas chronic treatment induces necrosis only after long treatment [25]. Accordingly, a significant increase in cell apoptosis was observed after few days of low dose of TNF treatment (Fig 2D, p<0.05), which accounts for cell proliferation reduction. The presence of PK 11195 in the medium with TNF has a very mild, but significant, effect on both cell proliferation and apoptosis (Fig 2C and 2D, p<0.05).

### Cell metabolism

**Fig 3:**
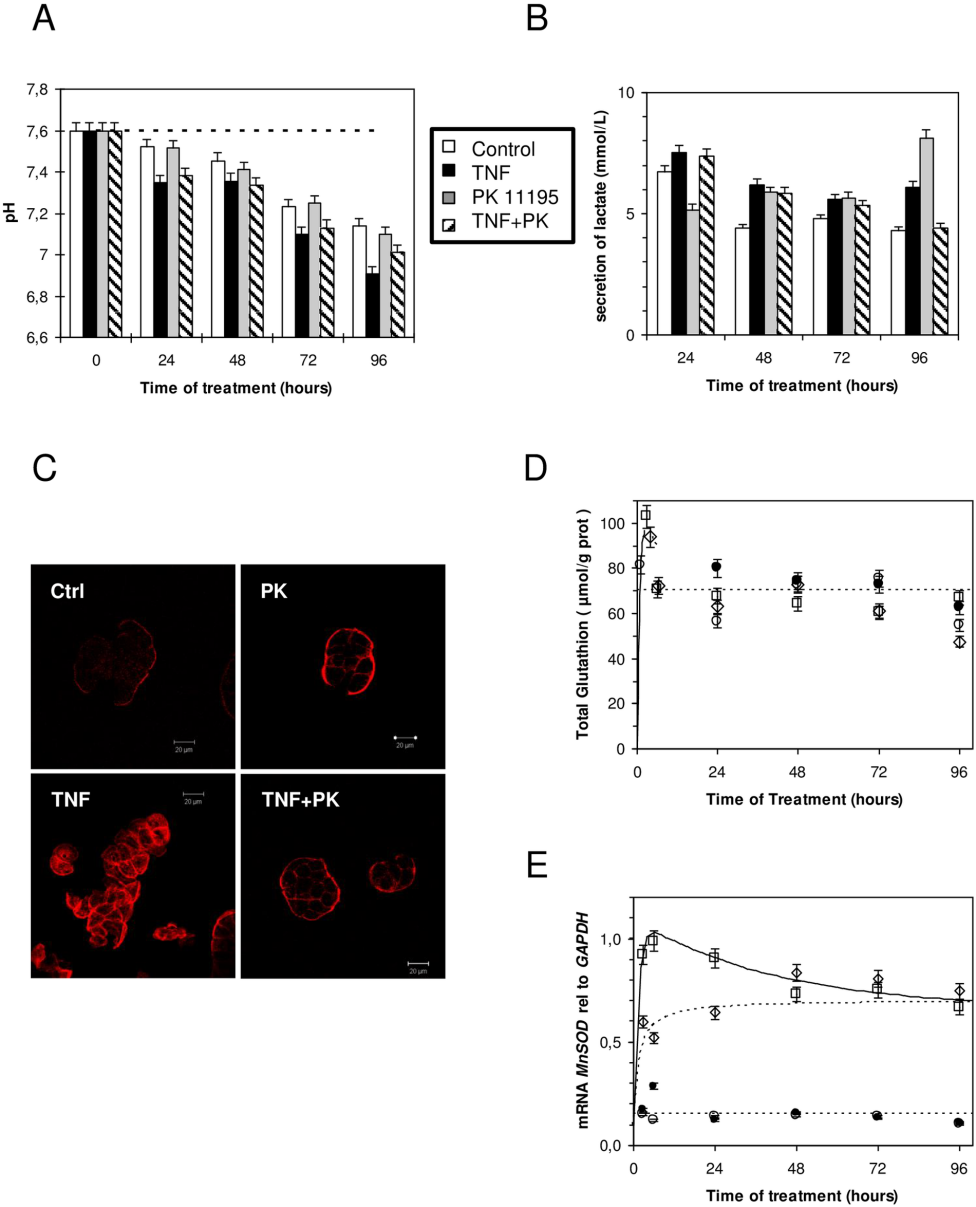
HT-29 cell metabolism upon TNF treatment. (A) Time course of cell culture medium pH change as a function of treatment (open bars, control; black bars, 10 ng/mL of TNF renewed daily; grey bars, 1 μM of PK 11195 treatment; dashed bars, 10 ng/mL of TNF and 1 μM of PK 11195, renewed daily). (B) Time-dependent lactate accumulation in cell culture medium; treatment as in panel A. (C) Actin stress fiber formation after 4 hours upon similar treatment as in panel A (control, upper left panel; 1 μM of PK 11195-treated cells, upper right panel; 10 ng/mL of TNF-treated cells, lower left panel; 10 ng/mL of TNF and 1 μM of PK 11195-treated cells, lower right panel). (D) Time-dependent total glutathione measurement; treatment as in panel A (closed circles, control; open squares, in the presence of 10 ng/mL of TNF daily replaced; grey triangles, 1 μM of PK 11195 replaced daily; grey diamonds, in the presence of 10 ng/mL of TNF and 1 μM of PK 11195, replaced daily). (E) Time course of *MnSOD* mRNA expression relative to *GAPDH*; treatment as in panel A (open circles, control; closed circles, in the presence of 1 μM of PK 11195, replaced daily; Open squares, in the presence of 10 ng/mL of TNF, replaced daily; open diamonds, in the presence of 10 ng/mL of TNF and 1 μM of PK 11195, replaced daily). The results are expressed as the means ± standard error of the mean.

**Fig 4:**
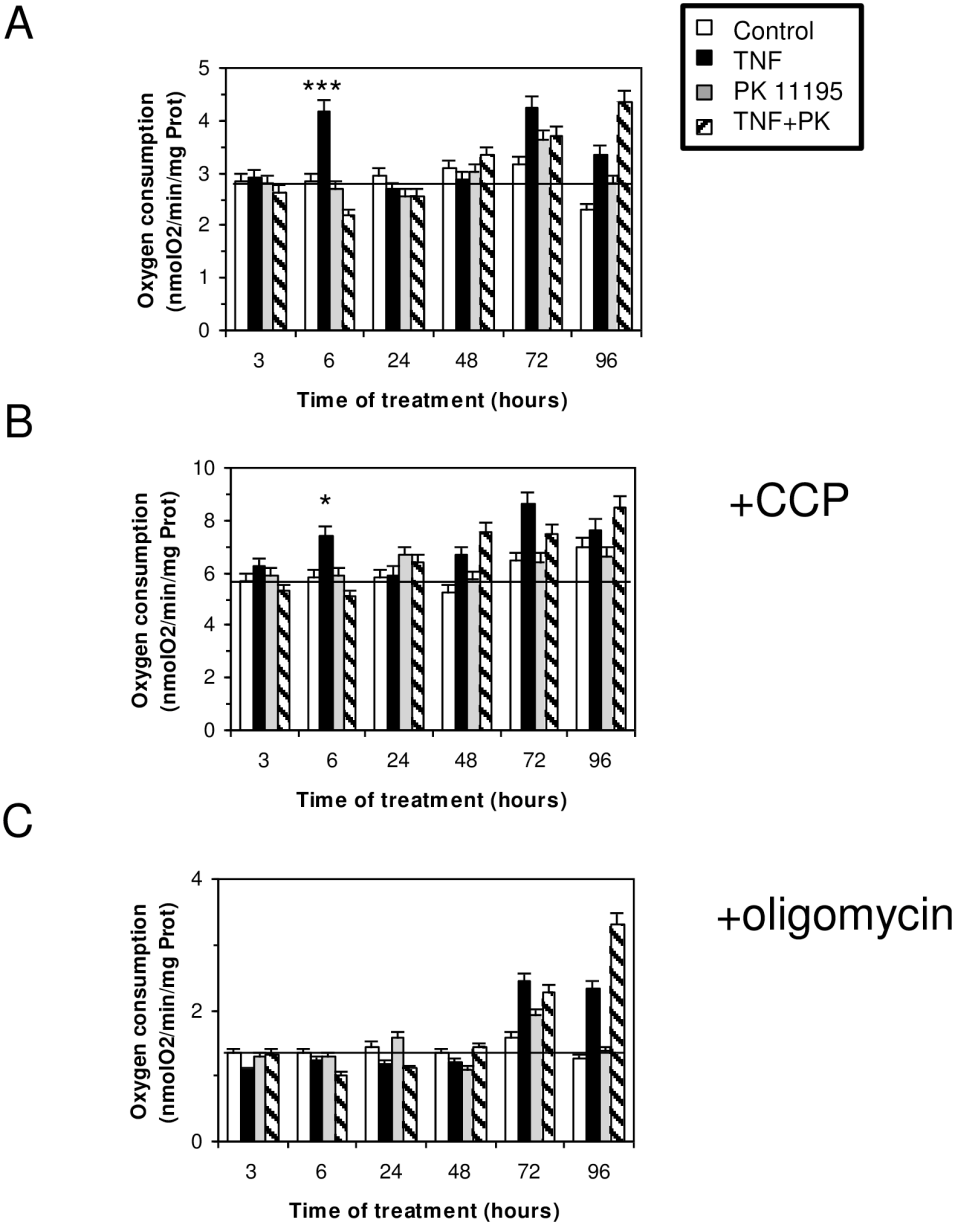
HT-29 cells’ oxygen consumption after different incubation times with 10 ng/mL of TNF. (A) Oxygen consumption in control cells (open bars), in TNF-treated cells (black bars), in 1 μM of PK 11195-treated cells (grey bars), and in cells treated simultaneously with 10 ng/mL of TNF and 1 μM of PK 11195 (dashed bars). (B) Oxygen consumption in the presence of 1 μM of *m*-Cl-CCP, a respiratory chain uncoupler. (C) Oxygen consumption in the presence of 10 μM of oligomycin, an ATP synthase inhibitor.

The addition of TNF in the culture medium of the HT-29 cells induced a clear acidification when compared to the control condition without TNF as revealed by the phenol color changing from red to yellow. This corresponded to 0.2 pH units between the culture with or without TNF after 24 h of cell culture (Fig 3A, p<0.001). This pH change suggests increased lactic acid production by cells treated by TNF (Fig 3B). It is well established that such secretion originates from an imbalance between glycolysis and pyruvate utilization by the mitochondria, resulting in pyruvate accumulation favoring lactate production and excretion [26]. Interestingly, this increase in lactate secretion, as well as the pH acidification observed in the presence of TNF, can be slightly reduced by adding PK 11195 in the medium (Fig 3A and 3B). It has been observed for years that, especially in cancer cell lines, glycolysis and lactate production are increased when there is a change toward anaerobic conditions or a deficiency in mitochondrial respiration [27], two conditions concomitant with the excessive production of ROS. In line with this, it has been described that TNF induces a ROS production mainly originating from mitochondria and that ROS are intermediates in TNF signaling [28,29]. In order to study this process, we first checked the TNF-induced actin stress fiber formation [30] using phalloidin–rhodamin, a fluorescent dye (Fig 3C). We showed that stress fibers are observed early after the beginning of HT-29 TNF treatment (4 hours), and that the presence of PK 11195 in the medium reduced such formation. We measured the total glutathione content, a key cellular antioxidant, which overproduction is triggered by TNF [31]. Fig 3D shows the change in glutathione status over the 4 days of treatment of HT-29 cells by TNF. A transient overproduction of glutathione is observed within the first 24 hours with a small but significant effect of PK 11195 added in the medium. We further measured whether the mitochondrial superoxide scavenger manganese superoxide dismutase (MnSOD) was overexpressed in the presence of TNF, as previously described [32–34]. Fig 3E shows that mRNA levels of MnSOD were strongly increased in HT-29 cells treated with TNF. Surprisingly, the first and rapid increase observed in the first 24 hours is strongly reduced by the presence of PK 11195. These data indicate that the TSPO drug ligand modulates superoxides produced by mitochondria. Fig 4A shows that a low dose of TNF (10 ng/mL) transiently increases oxygen consumption by HT-29 cells. This increase observed in the first hours following TNF treatment (6 hours, p<0.001) is completely blocked by the presence of PK 11195. The addition of a respiratory chain uncoupler (*m*-Cl-CCP) that dissipates the proton gradient, and thus reveals the maximal oxygen consumption, exhibits a similar profile to the oxygen consumption by HT-29 cells (Fig 4B). Thus, TNF does not uncouple the respiratory chain, but it increases global respiratory metabolism (more substrate enters in the chain). The addition of oligomycin, which blocks adenosine triphosphate (ATP) synthesis by the mitochondrial ATPase, reduces oxygen consumption and confirms that mitochondria are always well coupled (Fig 4C). The different effects of TNF treatment observed before and after 24 hours suggest a modulation in the role of TSPO in the inflammation related to ROS production.

### Mitochondrial morphology

**Fig 5:**
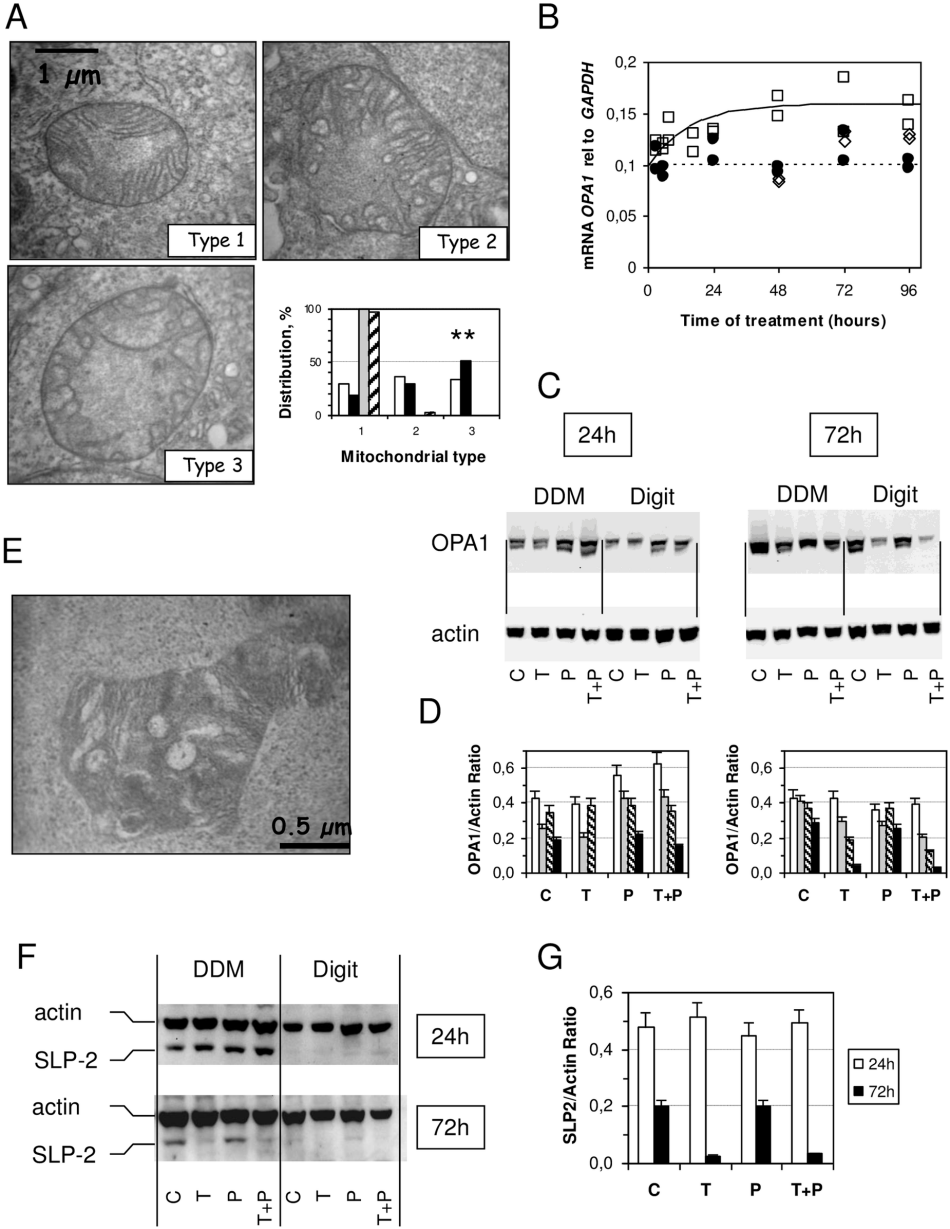
HT-29 cells’ mitochondrial ultrastructure and proteins change upon TNF treatment. (A) Electron micrographs of different types of encountered mitochondria [62] and their distribution in control cells (open bars), in TNF-treated cells (black bars), in 1 μM PK 11195-treated cells (grey bars), and in cells treated simultaneously with 10 ng/mL of TNF and 1 μM PK 11195 (dashed bars). (B) *OPA1* (optic atrophy 1) mRNA expression relative to *GAPDH* as a function of time of treatment with 10 ng/mL of TNF (open square), the simultaneous presence of TNF and PK 11195 (open diamonds), and controls without treatment (closed circle). (C) Western blot analysis of OPA1 protein in 0.1% dodecyl maltoside (DDM) and 0.1% digitonin (Digit)-solubilized mitochondrial membranes after 24 hours and 72 hours of treatment with 10 ng/mL of TNF [T], 1 μM of PK 11195 [P], and both 10 ng/mL of TNF and 1 μM PK 11195 [T+P] and controls [C]. (D) Densitometry evaluation of western blot of OPA1 protein in DDM (open and grey bars for long and short forms, respectively) and digitonin (dashed and black bars for long and short forms, respectively). (E) Electron micrograph of the mitochondria of HT-29 cells treated for 72 hours with 10 ng/mL of TNF. (F) Western blot analysis of the SLP-2 protein (stomatin-like protein-2) in DDM and Digit-solubilized mitochondrial membranes after 24 hours and 72 hours of treatment with TNF [T], PK 11195 [P], both TNF and PK 11195 [T+P], and controls [C], as in panel C. (G) Densitometry evaluation of western blot of SLP-2 in DDM-solubilized mitochondrial membranes. The results are expressed as the means ± standard error of the mean.

Since mitochondria are dynamic organelles [35], we then studied the ultrastructure of mitochondria upon 24 hours of TNF treatment. A profound modification of the mitochondrial ultrastructure was previously reported in L929 cell lines treated with TNF [36]. Three types of mitochondria can be differentiated in HT-29 cells (Fig 5A): mitochondria with well-formed cristae, or few or no stacked crystae (type 1); those with rounded up cristae (type 2); a third type (type 3) with swollen cristae accompanying an increase in mitochondrial size. The percents of these different types of mitochondrial ultrastructures are roughly equivalent in the non-treated HT-29 cells, but after 24 hours in the TNF-treated cells, a statistically significant increase in type 3 mitochondria is noticed (Fig 5A, p<0.01). Notably, the addition of PK 11195 in the medium with TNF prevents these mitochondrial ultrastructure changes and stabilizes well-formed cristae (type 1). This stabilization is due to PK 11195, since added alone it also favors the multilamellar stacked cristae type 1. Because OPA1, a mitochondrial protein involved in the fusion of mitochondria and their inner membranes is essential in stabilizing the cristae structure [35,37,38], we first quantified OPA1 mRNA expression in treated and non-treated HT-29 cells. Interactions between long and short forms of OPA1 are needed to stabilize the cristae, thus, any change in OPA1 composition leads to the remodeling of cristae.

We observed that the cristae rearrangement occured at the same time as OPA1 overexpression, as revealed by the increase in mRNA (Fig 5B), as well as by a change in the distribution of long and short forms of OPA1 protein, needed to stabilize the cristae [37,38], observed in Western blots (Fig 5C). Different mitochondrial membrane protein complexes can then be obtained according to the nature of the detergent used to solubilize them [19,39]. Mild detergent, such as digitonin (Digit), yields large protein complexes, whereas, dodecylmaltoside (DDM) yields smaller protein complexes. After 24 hours of TNF treatment, the amounts of long forms of OPA1 (95–92 kDa) are almost constant under all purification conditions. Short forms of OPA1 (88–81 kDa) are reduced, especially in digitonin-purified complexes, unless PK 11195 is presented simultaneously to TNF. When PK 11195 is present alone, short forms accumulate. After 72 hours of TNF treatment, the overall amount of OPA1 is reduced (Fig 5C) and the mitochondrial ultrastructure is modified (Fig 5E). This is consistent with the early increase levels of apoptosis observed after TNF addition. The matrix appears to be more condensed, the mitochondria swell with dense areas within the matrix. Such mitochondrial morphological changes have been previously observed in the stress-induced mitochondrial hyperfusion (SIMH) as an adaptive response against stress [16]. The maintenance of the long form of OPA1 is then ensured by a stomatin-like protein 2 (SLP-2), which is strongly associated with the mitochondrial inner membrane together with several other proteins like prohibitins and respiratory chain complexes [40]. Fig 5F shows that SLP-2 is stable after 24 hours of TNF treatment, but it decreases at 48 hours (not shown) and disappears at 72 hours of treatment. SLP-2 is clearly observed in complexes purified with DDM, but not in digitonin-purified ones, whereas OPA1 is observed in both. This suggests that DDM better solubilized TSPO-containing complexes than digitonin. The addition of PK 11195 does not prevent SLP-2 decrease in the presence of TNF. All of these observations suggest that the large complexes present in the mitochondrial membranes are modified during long-term treatment with TNF and that the capacity of the ligand to maintain this complex is effective only in the short term.

### Mitochondrial fusion

**Fig 6:**
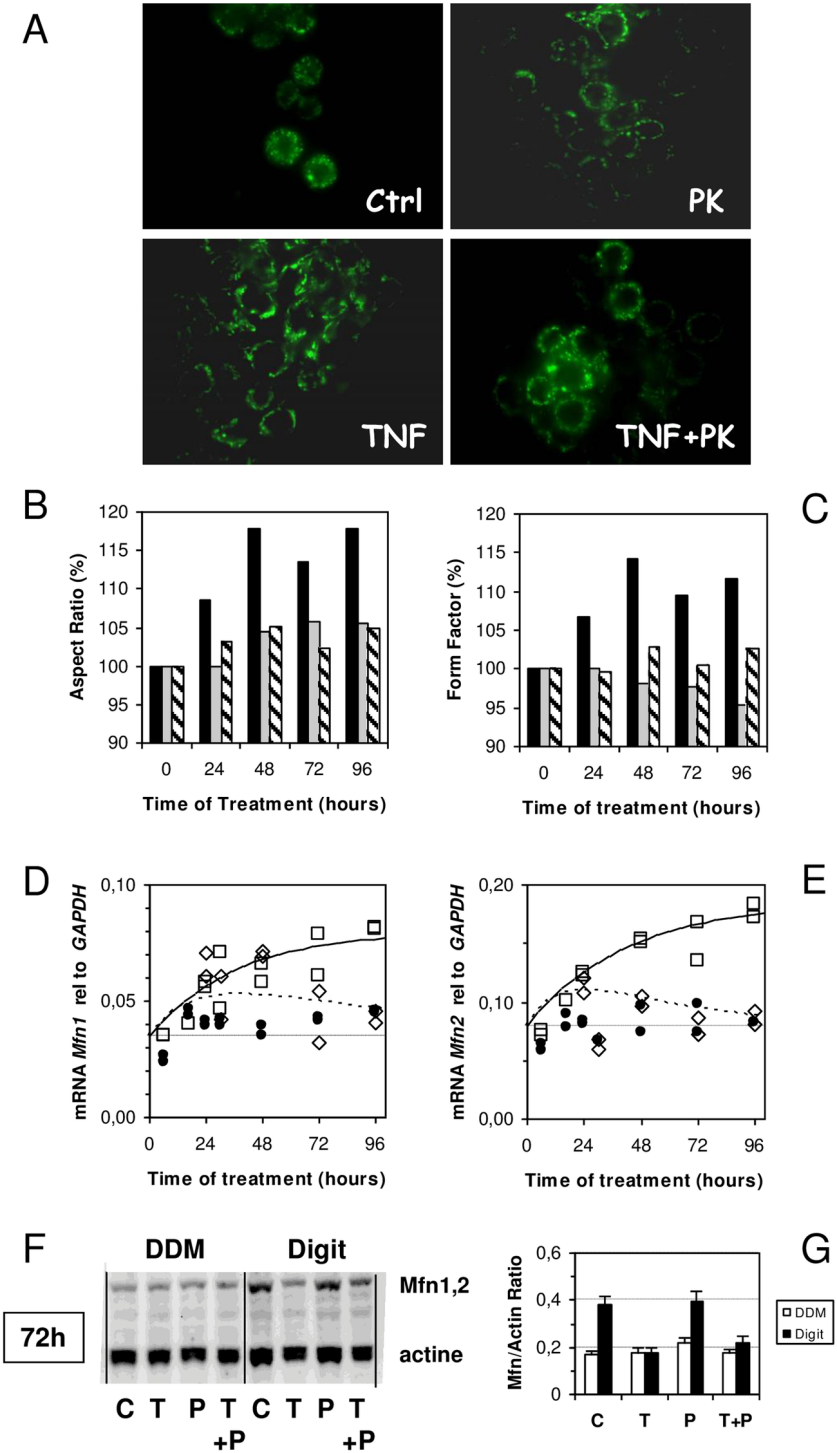
HT-29 cells’ mitochondrial networks and fusion proteins upon TNF treatment. (A), Representative images of HT-29 cells expressing green fluorescent protein (GFP) targeted to mitochondria in control and treated cells. (B and C) Quantitative analysis of mitochondrial structure using morphological parameters (Aspect Ratio and Form Factor) following Koopman et al. treatment. [23] At least 500–600 mitochondria were analyzed per condition and normalized to control conditions. The time course of the Aspect Ratio (B) and Form Factor (C) changes in TNF-treated cells (black bars), 1 μM of PK 11195-treated cells (grey bars), and cells treated simultaneously with 10 ng/mL of TNF and 1 μM of PK 11195 (dashed bars). mRNA expression of mitofusins (*Mfn1* and *Mfn2* in panels D and E, respectively) relative to *GAPDH* as a function of time of treatment with 10 ng/mL of TNF (open square), the simultaneous presence of TNF and PK 11195 (open diamonds), and controls without treatment (closed circle). (F) Western blot analysis of mitofusins (1 and 2) in DDM and Digit-solubilized mitochondrial membranes after 72 hours of treatment with TNF [T], PK 11195 [P], both TNF and PK 11195 [T+P], and controls [C]. (G) Densitometry evaluation of western blot of mitofusins. The results are expressed as the means ± standard error of the mean.

Known to interact with SLP-2 and OPA1, the mitofusins (Mfn1 and Mfn2) are possibly partners in these complexes [35]. Like OPA1, these proteins are large GTPases anchored in the outer mitochondrial membrane, and they are involved in mitochondrial fusion process [41–43]. HT-29 cells containing GFP targeted to the mitochondria shows that TNF treatment induces mitochondrial fusion that is prevented in the presence of PK 11195 (Fig 6A). The analysis of each individual mitochondrion (see Materials and methods) demonstrated that TNF treatment induces mitochondrial fusion, a conclusion strengthened by the increases of both AR-Aspect Ratio (Fig 6B) and the FF-Form Factor (Fig 6C). A comparison of the averaged values of the AR and FF of the mitochondria measured along several days of treatment by TNF shows that mitochondrial fusion takes place as early as 24 hours, being roughly maximal at 48 hours. This is in agreement with the mitofusins’ mRNA expression, which increases along with TNF treatment (Fig 6D and 6E, for Mfn1 and Mfn2, respectively). A decrease in Mfn1 and Mfn2 was observed in the complexes prepared with digitonin upon TNF treatment, whereas Mfn1 and Mfn2 do not seem to be modified in the DDM-purified complexes. This suggests the alteration of large complexes, which seems to be less important in the presence of PK 11195 (Fig 6F).

### TSPO in the mitochondria

**Fig 7:**
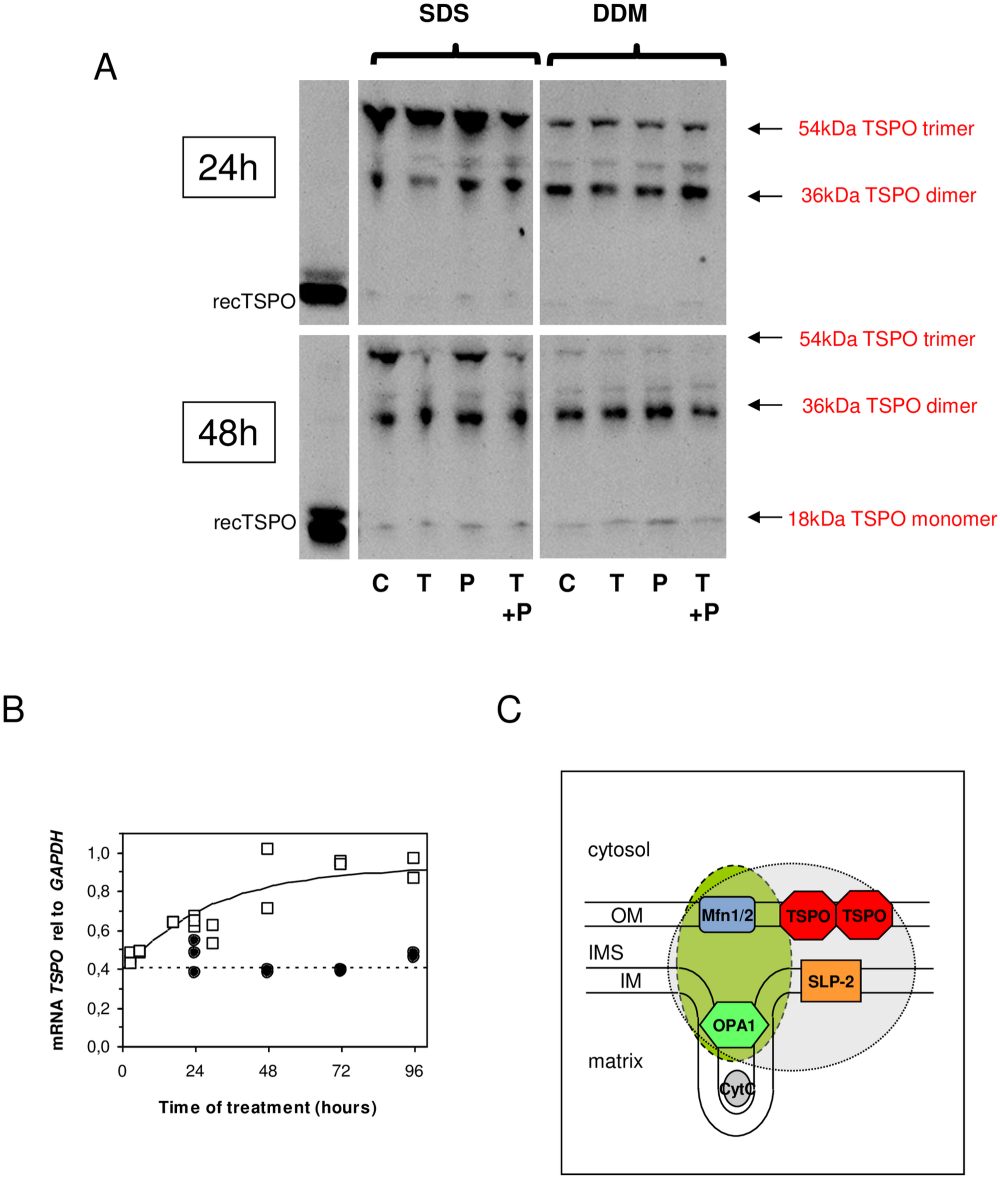
TSPO in HT-29 cells. (A) Western blot analysis of TSPO in SDS and DDM-solubilized mitochondrial membranes after 24 hours and 48 hours upon treatment with TNF [T], PK 11195 [P], both TNF and PK 11195 [T+P], and controls [C]. (B) *TSPO* mRNA expression relative to *GAPDH* as a function of the time of treatment with 10 ng/mL of TNF (open square) and controls without any treatment (closed circle). (D) Scheme of mitochondrial membrane proteins involved in different complexes revealed by partial solubilization with DDM (blue-colored circle) or digitonin (green-colored ellipse). The results are expressed as the means ± standard error of the mean.

Depending on the detergent used, two types of membrane complexes containing OPA1 and Mfn can be solubilized from the mitochondrial membrane, either containing SLP-2 (when using DDM) or not (when using digitonin). The regulation of these complexes seems different, as revealed by the mRNA and Western blot presented above. We therefore looked for the presence of the TSPO in these different complexes and its polymerization status. Indeed, it has been shown that in the presence of an excess of ROS, TSPO tends to form covalent polymers in Leydig and breast cancer cells as well as in reconstituted recombinant TSPO [44]. Fig 7A shows that this is also the case for HT-29 cells. In this instance, trimers and dimers are observed in SDS- and DDM-solubilizing conditions, but not in the presence of digitonin. Upon 24 hours of TNF treatment, the ratio trimer–dimer observed in SDS is displaced in favor of the trimer, which is indicative of the formation of covalent bonds, and is possibly favored by the presence of ROS (Fig 3). This change was not observed when TNF and PK 11195 are simultaneously present, suggesting protection by the TSPO drug ligand. At 48 hours of TNF treatment, we expected the same trend since the excessive production of ROS induced by TNF is still present. Surprisingly, this ratio was reversed (Fig 7A), and PK 11195 no longer affected this balance. This was also true after 72h treatment. The total amount of TSPO did not seem to change, but the trimers almost completely disappeared. The presence of reduced amounts of monomers, together with the large amount of dimer, suggested the *de novo* synthesis of TSPO. This is confirmed by the increase in transcription, which showed an increase in TSPO mRNA expression (Fig 7B). This indicates that TSPO turnover depletes trimers and generates dimers.

All of these observations suggest the existence of one complex that links TSPO in the outer membrane and OPA1 and SLP-2 in the inner membrane (Fig 7C); the two latter proteins are involved in the organization/stabilization of the inner membrane. This could explain how a disturbed TSPO conformation upon inflammation-favoring treatment, such as an increase of ROS, can modify the integrity of the TSPO-containing complexes. The modification observed in the large complex by OPA1 and Mfn appeared to be consequences of TSPO dysfunction. The TSPO ligand, PK 11195, is effective when protecting the complex against ROS overproduction for a short time period, reducing IL-8 cytokine production. However, it cannot fully protect against long-term inflammation treatment. Indeed, the increase in apoptosis observed at 72 hours–together with the disappearance of SLP-2, as well as the modification of the complexes, mitochondrial ultrastructure, and metabolism–suggest that the TSPO ligand is no longer able to prevent the inflammation.

### Role of TSPO in inflammation

**Fig 8:**
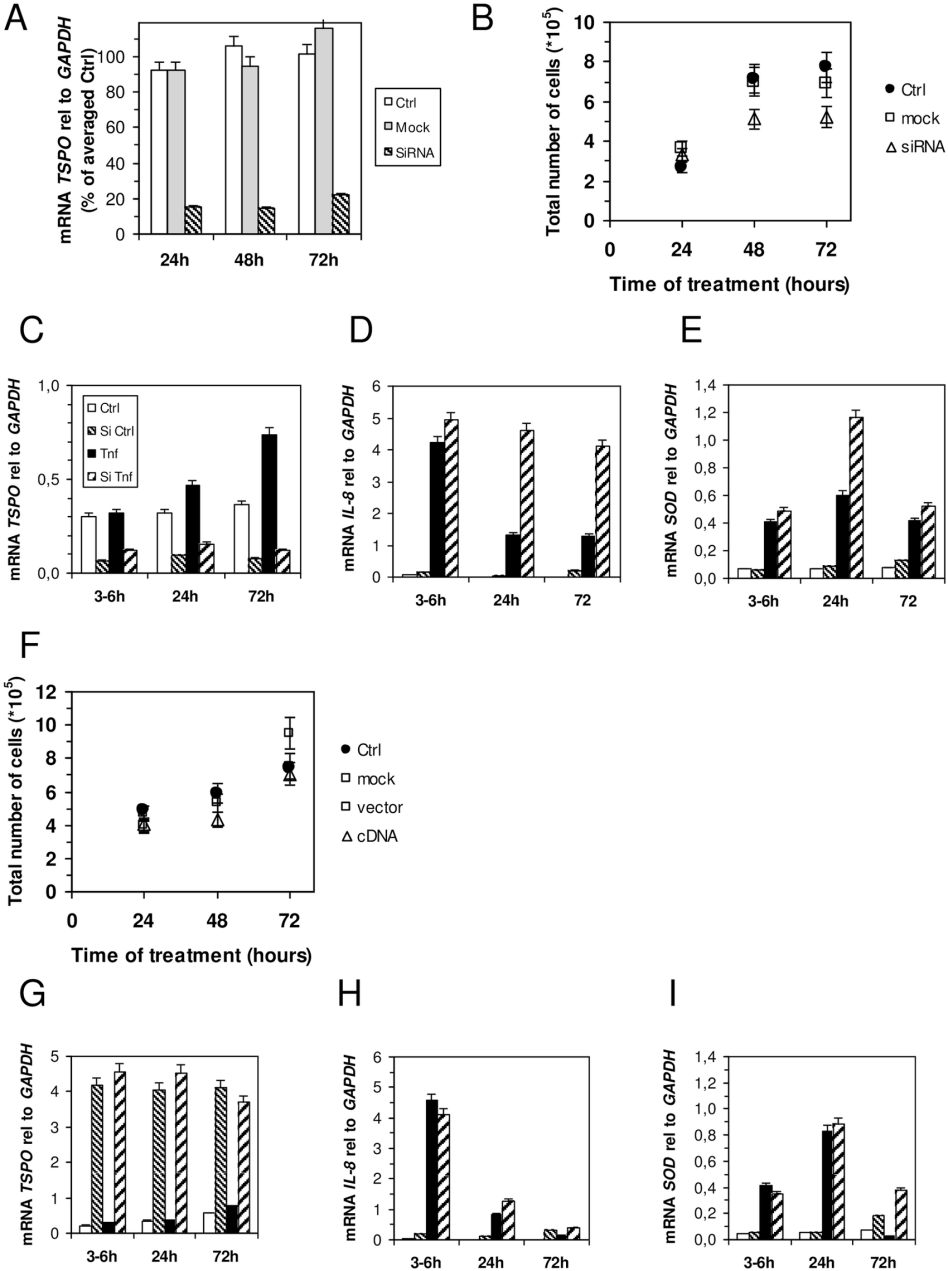
Effect of silencing (A–E) and overexpressing (F–I) TSPO in HT-29 cells. (A) Time dependence of TSPO mRNA expression in control cells (open bars), after mock transfection (grey bars) or siRNA-targeting TSPO transfection (dashed bars). (B) HT-29 cell proliferation (controls, black circles; mock-transfected, grey squares; and siRNA-targeting TSPO transfected, grey triangles). Time course of mRNA expression of *TSPO* (C), *IL-8* (D), and *MnSOD* (E) relative to *GAPDH* after 3–6 hours, 24 hours, or 72 hours of treatment with 10 ng/mL of TNF performed 24 hours after transfection by siRNA-targeting TSPO (controls, open bars; siRNA alone, short dashed bars; TNF-treated, black bars; siRNA and TNF-treated, long dashed bars). (F) HT-29 cell proliferation (controls, black circles; mock-transfected, grey squares; empty vector plasmid, open squares; and TSPO cDNA-containing vector, grey triangles) in the presence of TNF (nontransfected cells or siRNA-targeting TSPO transfected cells). Time dependence of mRNA expression of *TSPO* (G), *IL-8* (H), and *MnSOD* (I) relative to *GAPDH* after 3–6 hours, 24 hours, or 72 hours of treatment with TNF performed 24 hours after transfection by vector plasmid (controls, open bars; cDNA alone, short dashed bars; TNF-treated, black bars; cDNA and TNF-treated, long dashed bars). The results are expressed as the means ± standard error of the mean.

The inflammatory disorder induced by TNF appears to be a two-step process: 1) at a very early time (0–6 hours), large amounts of IL-8 are produced together with ROS; ROS can be regulated by the TSPO ligand, but not by IL-8. 2) Later on (within the first few days), stress induces mitochondrial morphology changes and protein turnover that lead to apoptosis, as observed after 3 days of TNF treatment. To better characterize the role of TSPO during this process, we first studied the effect of the absence of TSPO in HT-29 cells during inflammation. With this aim, we used siRNA–TSPO transfected cells. We obtained an 80% decrease in mRNA expression of *TSPO* at 24–72 hours after the transfection (Fig 8A). We noticed that upon 72 hours of treatment, the proliferation of siRNA-treated cells slows down (Fig 8B), which is in agreement with previous studies on breast cancer cell lines [45] this decrease was not seen in MA-10 and C6 glioma cells lines [46,47], or in HT-29 cells [48]. Thus, we treated the cells with TNF 24 hours after transfection and measured the mRNA expression of *TSPO*, *IL-8*, and *MnSOD* up to 72 hours (Fig 8C–8E). TNF still induced a small increase in *TSPO* mRNA expression (Fig 8C), presumably due to the 20% remaining mRNA. As we expected, the mRNA expression of *IL-8* increased in TSPO-depleted cells as early as 3–6 hours, and this observation is clearer at 24 and 72 hours of TNF treatment (Fig 8D). Similarly to *IL-8* mRNA, the *MnSOD* mRNA rapidly increased after a few hours, with the largest increase observed at 24 hours (Fig 8E). These data confirmed that TSPO was directly involved in inflammation in the early steps of the process, and that the effect of the ligand was directly linked to the presence of the protein. For the long-term treatment of siRNA–TSPO cells with TNF (72 hours), the mRNA of *IL-8* increased, but not that of *MnSOD* (Fig 8E). This might be due to the reduction in cell proliferation and the increase in cell death.

On the other hand, when the expression of TSPO was increased by transfecting HT-29 cells with cDNA, no change of cell growth was observed over several days (Fig 8F). The ten-fold overexpression of *TSPO* (Fig 8G) was found to be stable for several days. Under this condition, TNF treatment does not significantly induce *TSPO* mRNA changes (Fig 8G), whereas *IL-8* (Fig 8H) and *MnSOD* (Fig 8I) mRNA are overexpressed at a level similar to that of non-transfected cells.

Altogether, this study shows that in the absence of TSPO, inflammation is increased and antioxidant systems are induced, whereas overexpression does not counteract this effect.

## Discussion

**Fig 9:**
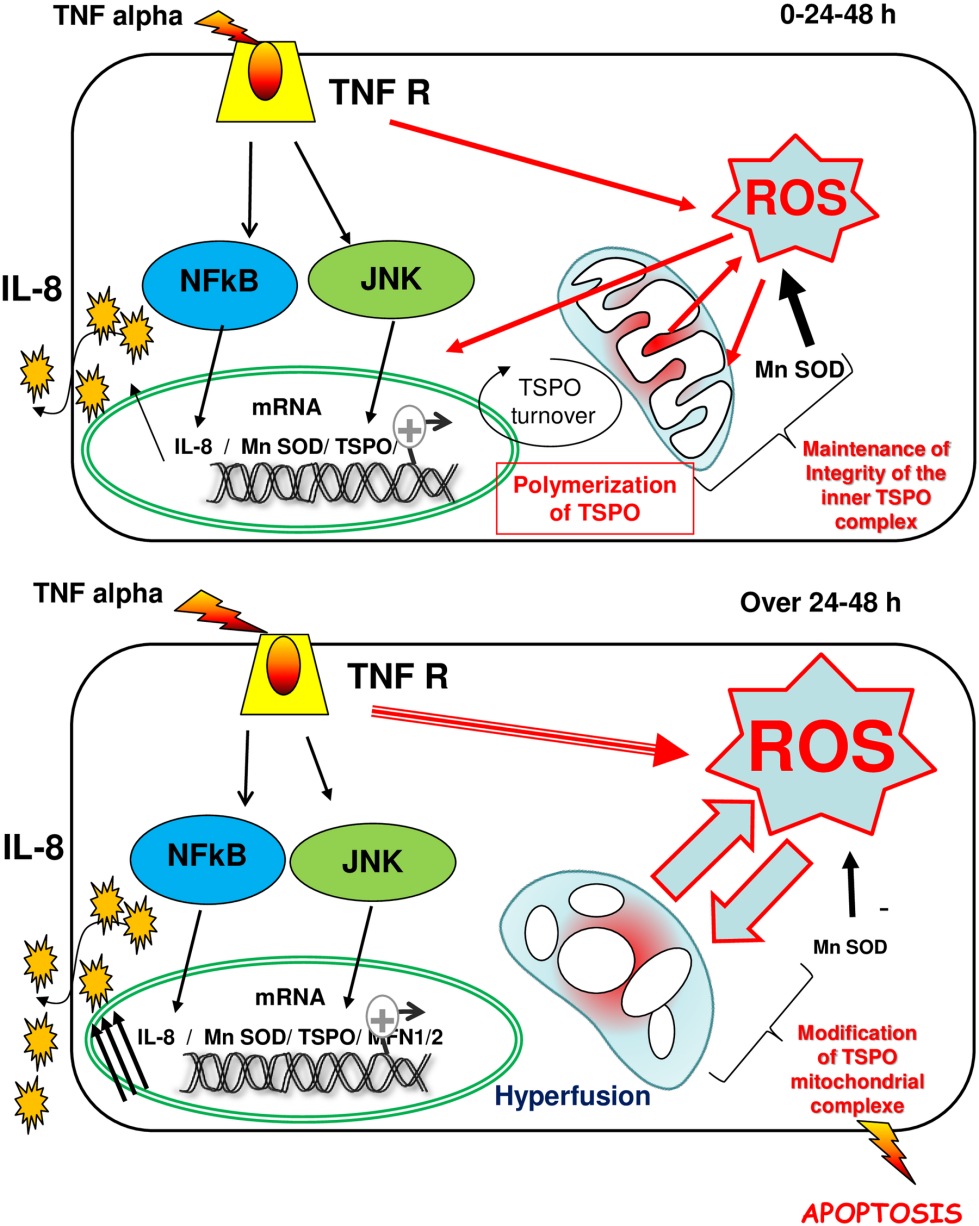
Modeling the involvement of TSPO upon TNF-induced inflammation. In the upper diagram, before 24 hours of treatment, TNF activates the production of IL-8 through NFκB signaling, as previously described [63]. Under this condition, ROS are produced and the antioxidant defenses (MnSOD) are activated simultaneously to TSPO transcription by the JNK pathway [52]. ROS induce the formation of TSPO polymers acting as ROS scavengers, and they induce an increase in TSPO turnover. This mechanism maintains the integrity of the mitochondria (inner ultrastructure membrane complexes) and favors regulation of the ROS production. In the lower diagram, after 24 hours, treatment with TNF causes an overflow of antioxidant defenses together with sustained TSPO turnover, leading to the dysfunction of mitochondrial membrane complexes responsible of the disruption of inner mitochondrial integrity; the apoptotic cascade is then activated, leading to cell death.

In the gastrointestinal tract, basal inflammation is a tightly regulated process. In IBD, such as ulcerative colitis and Crohn’s disease, inflammation becomes out of control. This affects mitochondria bioenergetics, increasing ROS and/or succinate [49,50]. The deleterious conditions in the intestinal epithelial barrier have, indeed, multiple consequences leading to death signaling. In previous studies, it has been shown that an overexpression of TSPO was associated with poor prognostic survival in patients affected with stage III colorectal cancer [51]. Although TSPO has been linked to apoptosis or cell-cycle arrest, the potential role of this protein in this disease is not fully understood. In this study, we tried to identify the possible actors involved in this process. In particular, we analyzed the role of TSPO in inflammatory conditions using HT-29 colonic cell lines, which were previously described to express mitochondrial TSPO [13] and to be responsive to TNF-induced inflammation [24]. A 4-days TNF treatment is deleterious for these cells, leading to apoptosis. The time course of the inflammation induced by TNF (the secretion of proinflammatory cytokines, as well as regulation of the metabolism, mitochondria ultrastructure, and network), indicated that two stages occurred in this process (Fig 9).

At the early stages (up to 24 hours), the increased IL-8 expression previously reported indicates that transcription and the production of cytokines are among the first consequences of inflammation. In keeping with this, it has been suggested that excess IL-8 secretion would stimulate parallel signaling pathways. In particular, this would include JNK signaling, known to activate transcription factor AP-1, ultimately leading to the increased transcription of TSPO [52]. In line with this observation, we noticed an overexpression of *TSPO* mRNA at 24 hours, but interestingly enough, only the trimeric form of the protein was increased. A polymerization of TSPO has been previously observed in correlation with ROS production [44]. Noticeably, we observed *MnSOD* overexpression, indicative of increased mitochondrial ROS production [32–34]. The presence of PK 11195 (a specific high-affinity TSPO ligand) prevents the disappearance of the TSPO dimer, suggesting a stabilizing effect of the ligand in agreement with structural data gained on the monomer [53,54]. Stabilization of the dimeric form might be responsible of the maintenance of the mitochondrial ultrastructure and network, illustrated by electron microscopy and confocal imaging studies in the presence of PK 11195. These results suggest that TSPO plays a role in the maintenance of the mitochondrial ultrastructure, with consequences on the regulation of mitochondrial metabolism. In particular, we observed a decrease in fiber stress among inflammatory cells in the presence of PK 11195. Accordingly, in TSPO siRNA-transfected cells, we observed an increase of both *IL-8* and *MnSOD* mRNA after 24 hours of TNF treatment, confirming a link between ROS regulation and TSPO. Thus, it appears that upon ROS production, TSPO forms polymers acting as ROS scavengers, in order to maintain mitochondrial membrane functional. Ultimately increasing TSPO turnover may counteract the deleterious effect induced by the excessive ROS production. ROS production was indeed used as a target to fight colitis induced by dextran sulfate sodium (DSS) in the mouse [55]. Moreover, it has been recently suggested that upon inflammation, ROS overproduction occurs when succinate deshydrogenase (SDH) drives reverse electron transfer through mitochondrial complex I [49,50]. We hypothesize that TSPO is involved in the stability of the mitochondrial ultrastructure and of mitochondrial TSPO-containing complexes directly linked to the regulation of the metabolism that produces ROS. TSPO would then act as a scaffold protein within a large complex with other scaffold proteins such as OPA1, SLP-2, and Mfn1/2, controlling the integrity of the inner mitochondrial membrane, especially of the cristae, which is important for the respiratory chain function [56]. Thus, at an early stage, TSPO would contribute to the maintenance of the mitochondrial structure counteracting inflammation by maintaining cell metabolism.

With longer treatments with TNF (after 24 hours), ROS production increased and metabolism was affected. In an excessive ROS environment, TSPO has been shown to form polymers through covalent bonds [44]. Our data showed a depletion in the trimeric form of TSPO. We could speculate that the degradation of the polymerized TSPO is taken to a specialized signaling pathway involved in mitochondrial protein quality control called MAD (Mitochondrial-Associated Degradation) [57]. In this system, damaged proteins particularly by oxidative stress, are accessed to the proteasome after ubiquination at the OMM. In parallel with this, the observed turn over would be important to maintain the role of TSPO in the stability and integrity of the mitochondria. This dysregulation appeared to affect the TSPO-containing complexes and it further increased ROS production, ultimately causing apoptosis signaling. Interestingly, not all TSPO-containing complexes were modified in the same manner. Using selective detergent solubilization (digitonin and DDM), we observed that dimeric TSPO is present in the complexes solubilized by DDM and absent in those solubilized by digitonin. However, both complexes are modified upon long-term treatment with TNF, which is in agreement with the role of the proteins studied herein (OPA1, SLP-2, Mfn1/2) in the stability of the cristae. Finally, the destabilization of the mitochondrial inner membrane appeared to favor cell death. In keeping with this, the voltage-dependent anion channel (VDAC), which is located similarly to TSPO in the outer membrane, has been recently shown to participate in the ROS-mediated inhibition of mitochondrial quality control [58]. However, our study highlighted that TSPO only afforded short-term protection, as silencing and overexpression studies (siRNA and cDNA) demonstrated that long, chronic treatment abolished this protective effect. The results obtained from TSPO expression studies differed from the ones in previous studies in microglial cells, where overexpression of TSPO decreased the production of proinflammatory cytokines upon lipopolysaccharide treatment, while TSPO knockdown had the opposite effect [59]. These different effects might be linked to the nature of the used cell lines. We can hypothesize that in this system, the role and regulation of TSPO in the complex might be different. Indeed, the intracellular signaling involved in the inflammation process must be different between the brain and intestine, as well as in the existing complexes. Moreover, the opposite effect of TSPO expression on cell viability might originate from the use of different experimental conditions to delete TSPO, such as antisense RNAs [48,60].

Upon treatment of chronic inflammation, another mechanism of defense for the mitochondria appeared to take place; this was similar to an hyperfusion mechanism [16,59]. This process just preceded apoptosis. Nevertheless, no hyperfusion during longer times was observed in cells treated with TNF and PK 11195. One explanation would be that PK 11195 did not prevent inflammation, but it only delays the deleterious effect of TNF treatment by regulating the conformation (folding or polymerization) of TSPO in the cells. This is reminiscent of the effect of PK 11195 observed in the DSS-treated rat [11]. Moreover, overexpression of TSPO did not result in any recovery from the inflammatory phenotype, stressing the fact that folding, and not the amount of TSPO, is required to counteract inflammation. It thus appears that the integrity of the protein is important, but not sufficient, to prevent inflammation. Presumably, the TSPO protein is not sufficient by itself, but it is associated with additional proteins within a membrane complex that are active and control mitochondrial ROS production.

## Conclusion

We show herein that TSPO is a scaffold protein that is part of those mitochondrial membrane complexes involved in the maintenance of the integrity of mitochondrial cristae. As a member of these complexes, TSPO participates in a number of processes, such as the regulation of the mitochondria network and metabolism. Under inflammatory conditions, TSPO-containing complexes take part in the global mechanism of anti-inflammatory processes through the regulation of ROS production, which is also described as important to prevent metastasis [61]. Future work should focus on comprehending the formation and the actual composition of such complexes, hoping to determine the exact mechanism by which these TSPO complexes control mitochondrial ROS production upon chronic inflammation.
